# EV-associated non-coding RNAs in paediatric asthma airway remodelling: current mechanistic evidence and therapeutic perspectives

**DOI:** 10.3389/fphar.2026.1820574

**Published:** 2026-05-07

**Authors:** Yaling Liu, Dan Wang, Hu Gao

**Affiliations:** 1 WCSUH-Tianfu ⋅ Sichuan Provincial Children's Hospital, Meishan, Sichuan, China; 2 West China Second University Hospital, Sichuan University, Chengdu, China

**Keywords:** airway remodelling, circRNA, EV-associated ncRNA, exosomes, extracellular vesicles, lncRNA, microRNA, paediatric asthma

## Abstract

Paediatric asthma is a chronic inflammatory airway disease that can cause airway swelling, excessive mucus secretion, and increased smooth muscle tone, leading to airway narrowing and respiratory distress. Airway remodelling involves a series of pathophysiological alterations, including airway epithelial injury, proliferation of mucous glands and goblet cells, subepithelial fibrosis, proliferation and migration of airway smooth muscle cells, and airway epithelial-mesenchymal transition. These complex processes significantly increase airway resistance and reactivity, forming a crucial pathological basis for refractory asthma. With advances in molecular biology, non-coding RNAs such as microRNAs (miRNAs), long non-coding RNAs (lncRNAs), and circular RNAs (circRNAs) have garnered extensive attention due to their pivotal roles in gene expression regulation. Extracellular vesicles (EVs), as membrane-bound particles released by cells, can carry various bioactive molecules including non-coding RNAs, playing a crucial role in intercellular communication and influencing the functional state of recipient cells. In diseases such as asthma, non-coding RNAs participate in regulating processes including airway remodelling, emerging as potential diagnostic biomarkers and therapeutic targets. This narrative review summarises current evidence on EV-associated non-coding RNAs in paediatric asthma airway remodelling, noting that most cited studies used operational EV terminology rather than biogenesis-specific “exosomes” because endosomal origin was not experimentally validated in most cases. Current mechanistic evidence is strongest for miRNA-related studies, whereas direct evidence involving EV-associated lncRNAs and circRNAs in paediatric airway remodelling remains limited. The review therefore focuses on currently available mechanistic evidence, translational relevance, and major knowledge gaps.

## 1 Introduction

Asthma is a chronic inflammatory airway disease primarily characterised by airway hyperresponsiveness, that is, bronchoconstriction triggered by environmental or immunological stimuli. Its pathophysiology involves chronic inflammation, immune dysregulation, and airway remodeling (Hammad et al., 2025). Microbial exposure, infection, air pollution, as well as stress, allergens, and tobacco smoke may all be risk factors for asthma. Asthma can occur at any age, but it is especially common in children. Research indicates that 23 million children worldwide are affected by asthma (Zhang and Zheng, 2022). Surveys conducted in 1990, 2000, and 2010 across 16 Chinese cities on the prevalence of paediatric asthma among children aged 0–14,showed that the asthma prevalence rates among urban children were 0.96%, 1.66%, and 2.38% respectively, indicating a significant upward trend (Liu et al., 2015). At present, the cornerstone of pharmacological treatment for paediatric asthma is the use of inhaled corticosteroids (ICS), with long-acting β_2_-agonists (LABA) added when clinically indicated to improve symptom control. Also, encouraging patients to engage in regular exercise and other non-pharmacological interventions is also part of the comprehensive management plan (Global Initiative for Asthma, 2025). Although current drug therapies are effective, they still have limitations in clinical practice. Long-term use of inhaled corticosteroids and similar medications in pediatric patients may induce adverse reactions (Ma et al., 2025), such as bone density reduction and growth retardation. Although early inhaled corticosteroids (ICS) can effectively control asthma symptoms and reduce the risk of acute exacerbations, their use cannot reverse the underlying disease process of asthma (Szefler, 2007). Therefore, exploring novel therapeutic approaches is necessary to fulfill clinical treatment demands.

Airway remodelling is underpinned by long-term chronic airway inflammation (Yang et al., 2025), and may involve pathological changes such as epithelial fragility, goblet cell hyperplasia, enlargement of submucosal mucous glands, angiogenesis, increased deposition of airway wall matrix, increased mass of airway smooth muscle (ASM), thickening of the airway wall, and abnormal elastic fibres. These alterations markedly increase airway resistance and hyperresponsiveness, forming a crucial pathological basis for refractory asthma (Sahnoon et al., 2025; Yuan et al., 2023). Airway remodelling is more evident in persistent or severe asthma, although selected structural changes have also been reported in some patients with earlier or milder disease (Malmstrom et al., 2011). Not all inflammatory pathways active in paediatric asthma necessarily lead to fixed structural airway changes; therefore, this review prioritises mediators and pathways more directly linked to remodelling-related structural alterations, rather than inflammatory mechanisms common to paediatric asthma more broadly. Currently, the regulatory mechanisms of airway remodelling primarily focus on two key aspects: signalling pathways and regulatory targets. Signalling pathways involve phosphoinositide 3-kinase (PI3K)/Protein Kinase B (Akt), Nuclear Factor-κB (NF-κB), Transforming Growth Factor-β1 (TGF-β1)/Smads, and Mitogen-Activated Protein Kinase (MAPK) pathways (Yap et al., 2019), whilst regulatory targets encompass microRNAs (miRNAs), long non-coding RNAs (lncRNAs), and circular RNAs (circRNAs) (Yuan et al., 2025). Therefore, how to control airway remodeling in the early stages remains a key focus of current and future research.

Extracellular vesicles (EVs) are heterogeneous membrane-bound particles released by cells, among which exosomes represent a subtype of endosome-derived vesicles (Chen et al., 2025). They are present in multiple body fluids, including blood, urine, and saliva, and carry diverse molecular cargo such as non-coding RNA, messenger RNA, proteins, and lipids (Zhang L. et al., 2025). In this review, the term “EVs” is used unless endosomal origin has been experimentally demonstrated. Among the diverse EV cargo components, non-coding RNAs (ncRNAs) are of particular interest because they regulate gene expression without being translated into proteins. This category includes microRNAs (miRNAs), circular RNAs (circRNAs), and long non-coding RNAs (lncRNAs).

EVs serve as carriers for intercellular material transfer, delivering regulatory cargo such as ncRNAs to recipient cells and thereby contributing to intercellular communication; they have also been associated with a range of pulmonary disorders, including asthma, pulmonary fibrosis, lung cancer, and acute lung injury (Lai et al., 2023; Purghe et al., 2021). In recent years, a growing number of studies have suggested that EV-associated non-coding RNAs may participate in remodelling-relevant processes in childhood asthma through effects on airway epithelium, airway smooth muscle, and extracellular matrix homeostasis. Among ncRNA subclasses, current mechanistic evidence is strongest for miRNA-related studies, whereas direct evidence involving EV-associated lncRNAs and circRNAs in paediatric airway remodelling remains comparatively limited. Accordingly, this review first outlines airway remodelling in paediatric asthma and then discusses how EV-associated ncRNAs may participate in these processes. Where direct evidence for EV-mediated transfer is unavailable, relevant ncRNA studies are interpreted cautiously as mechanistic context rather than definitive proof of EV-dependent intercellular communication, with particular emphasis on mechanistic relevance, translational potential, and current limitations.

## 2 Literature search and selection strategy

This article should be regarded as a narrative review rather than a formal systematic review. Literature was identified through searches of PubMed, Web of Science, and Google Scholar using combinations of keywords such as “paediatric asthma”, “airway remodelling”, “extracellular vesicles”, “exosomes”, “microRNA”, “lncRNA”, “circRNA”, and “EV-associated ncRNA”. Relevant English-language articles published up to 2025 were included, with priority given to studies reporting EV-ncRNA in asthma models or clinical samples. No formal inclusion/exclusion criteria, PRISMA flow diagram, or risk-of-bias assessment were applied, as this is a narrative synthesis aimed at summarising current evidence and highlighting potential mechanisms rather than performing a quantitative meta-analysis.

## 3 Overview of extracellular vesicles and their non-coding RNAs

### 3.1 Structural and functional characteristics of extracellular vesicles

Small extracellular vesicles (sEVs), often operationally defined as vesicles within the size range of approximately 30–150 nm, are sometimes referred to as “exosomes” when endosomal origin is demonstrated (Jeppesen et al., 2024). However, in many experimental studies, vesicles are classified primarily according to size, isolation strategy, or marker enrichment rather than direct validation of biogenesis. For this reason, the term “small EVs” is often more appropriate unless endosomal origin has been clearly established. Extracellular vesicles are commonly discussed as including small EVs, microvesicles, and apoptotic bodies. Although these categories are conceptually linked to different biogenetic routes, their experimental discrimination remains challenging because vesicle populations may overlap in size, composition, surface markers, and isolation characteristics (Kimiz-Gebologlu and Oncel, 2022). Therefore, these categories should be interpreted as useful operational frameworks rather than as always unequivocally separable populations. Most human cells can release extracellular vesicles, which are widely distributed in various bodily fluids, including cerebrospinal fluid, saliva, milk, lymph, bile, blood, and urine (Kalluri and LeBleu, 2020). EV membranes comprise a phospholipid bilayer that protects internal molecules from extracellular degradation. Their membranes contain proteins involved in EV recognition, localisation, trafficking, and interactions with recipient cells, while their luminal cargo includes proteins, DNA, RNA, lipids, and regulatory ncRNAs such as miRNAs and lncRNAs (Raposo and Stoorvogel, 2013).

**FIGURE 1:**
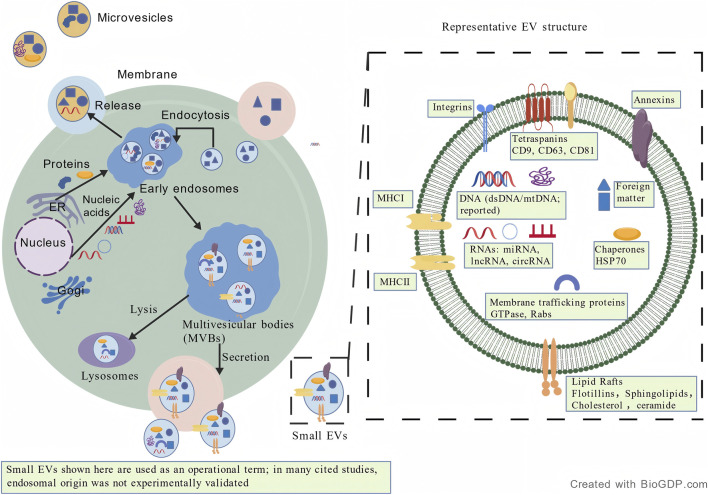
Schematic diagram of EV biogenesis and representative molecular composition.

Historically, the term “exosome” emerged from studies describing vesicles released through the endosomal pathway (Trams et al., 1981; Harding et al., 1983; Johnstone et al., 1987). For the purposes of this review, however, the most relevant aspect of EV biology is their ability to package, protect, and transfer ncRNAs to recipient cells, thereby influencing downstream signalling and cellular phenotypes relevant to airway disease. The biogenesis and representative molecular composition of EVs are illustrated in Figure 1.

Left panel: Overview of cellular pathways involved in EV release, including endocytosis, early endosomes, membrane budding, and lysosomal degradation. Endocytosed material is shown progressing from early endosomes to multivesicular bodies (MVBs), which may either undergo lysosomal degradation or contribute to the release of small EVs. Microvesicles are shown as vesicles formed by outward budding of the plasma membrane. Right panel: Representative structure of an EV showing commonly reported components, including tetraspanins (CD9, CD63, CD81), integrins, annexins, MHC molecules, chaperones, membrane trafficking proteins, lipid raft-associated lipids, and nucleic acids such as miRNAs, lncRNAs, circRNAs, and reported DNA species. This is a simplified schematic for illustrative purposes. Cargo loading is selective and multi-step; not all molecules are directly transported from the endoplasmic reticulum or nucleus to endosomal compartments. Small EVs shown here are used as an operational term, and in many studies included in this review, vesicle origin was not experimentally validated. Apoptotic bodies are not shown because they are generally larger and less relevant to the ncRNA-focused scope of this review. Created with BioGDP.com.

### 3.2 Classification of non-coding RNAs

#### 3.2.1 MicroRNAs

MicroRNAs constitute a class of small non-coding RNAs comprising 17 to 24 nucleotides, capable of regulating a range of biological processes. Beyond their intracellular functions, microRNAs may be encapsulated within EVs and secreted extracellularly. Circulating via EVs, they are subsequently internalised by neighbouring or distant cells, thereby modulating the functions of recipient cells (Guo et al., 2025). The target genes of miRNAs are mRNAs. They exert regulatory effects by binding to complementary sequences in the 3′untranslated region (UTR) of mRNAs, thereby degrading target mRNAs or inhibiting mRNA translation into proteins. Beyond EV-mediated encapsulation, certain miRNAs may also be loaded into high-density lipoproteins (Misir et al., 2025), while others can bind to AGO2 proteins on the exterior of vesicles (Arroyo et al., 2011). All three modes confer protection against degradation, ensuring miRNA stability. The loading of miRNAs into EVs is a regulated process, although the specificity of sorting mechanisms may vary across different EV subtypes. Multiple studies have proposed several mechanisms involved in miRNA sorting into EVs, including the neutral sphingomyelinase 2 (nSMase2)-dependent pathway, RNA-binding protein-related pathways, miRNA 3′-end sequence-dependent sorting, and microRNA-induced silencing complex (miRISC)-related mechanisms (Colombo et al., 2014; Kumar and Deep, 2020; Santangelo et al., 2016). Owing to their transportability, EV-associated miRNAs have garnered significant attention for their role in cellular communication.

#### 3.2.2 Long non-coding RNA (lncRNA)

First reported in 1990, lncRNA constitutes a class of non-coding RNA exceeding 200 nucleotides in length and is widely distributed across eukaryotic organisms (Quinn and Chang, 2016). Although lncRNAs do not encode proteins, they share certain biological similarities with mRNAs. Most lncRNAs are transcribed by RNA polymerase II and undergo capping and polyadenylation. However, while mRNAs require translation into proteins to fulfil specific cellular functions, lncRNAs themselves constitute functional units (Bridges et al., 2021), and lncRNAs are present in both the nucleus and cytoplasm. Within the nucleus, they regulate transcriptional programmes through chromatin remodelling and interactions, and establish the spatial organisation of nuclear compartments via scaffolding. In the cytoplasm, lncRNAs mediate post-transcriptional control of signalling pathways, translational processes, and gene expression. lncRNAs can be selectively packaged into EVs, circulating to nearby or distant cells to exert regulatory effects. They play a crucial role in both innate and adaptive immunity, including regulating immune system development, activation, and homeostasis (Mattick et al., 2023).

#### 3.2.3 circRNA

circRNA represents a unique class of endogenous non-coding RNA molecules discovered over forty years ago. EVs can encapsulate functional circRNA, transporting it from progenitor cells to target cells where it performs its functions. Similar to linear mRNA, circRNA originates from linear precursor mRNA transcribed by RNA polymerase II and is generated through reverse splicing of linear RNA (Muhammed et al., 2025). It is characterised by the absence of a 5′cap structure and 3′polyadenylate tail, presenting as a covalently closed circular structure (Qiu et al., 2018). As research advances, circRNA has emerged as a novel class of non-coding RNA, opening new avenues of investigation. Studies reveal circRNA broadly regulates human physiological and pathological processes, playing a significant role in modulating immune cell function. Four primary regulatory mechanisms exist: ①Acting as miRNA sponges. circRNAs contain miRNA-binding sites, blocking miRNA interaction with the 3′untranslated regions of target mRNAs to regulate mRNA expression (Zheng et al., 2016). ②Regulation of gene transcription. circRNAs are distributed in both the cytoplasm and nucleus, though predominantly found in the cytoplasm, potentially participating in transcriptional regulation of parental cell genes (Zeng et al., 2017). ③circRNA interacts with proteins. circRNA can bind to various proteins to form circular RNA-protein complexes, thereby regulating protein function, translation, and subcellular localisation (Li et al., 2015). ④circRNA can be translated into peptides or proteins. Certain circRNAs encode specific cellular stress responses and undergo translation (Legnini et al., 2017).

## 4 Overview of airway remodelling in paediatric asthma

### 4.1 Morphological mechanisms of airway remodelling

#### 4.1.1 Proliferation and migration of airway smooth muscle cells (ASMCs)

The proliferation and migration of airway smooth muscle cells (ASMCs) constitute one of the hallmark events of airway remodelling (Dekkers et al., 2009). In asthma, ASMC phenotype and function are regulated by inflammatory mediators, cytokines, and growth factors. Among these, TGF-β, PDGF-BB, VEGF, and MMP-related signalling are particularly relevant to ASMC proliferation, migration, and airway wall thickening (Shin et al., 2009; Camoretti-Mercado and Lockey, 2021). These changes contribute to luminal narrowing, impaired ventilation, and increased airway hyperresponsiveness (Listyoko et al., 2024; Zhang W. et al., 2023).

#### 4.1.2 Epithelial-mesenchymal transition (EMT)

Epithelial-mesenchymal transition (EMT) is an important process in asthma airway remodelling, in which airway epithelial cells acquire mesenchymal-like characteristics (Zhang B. et al., 2025). EMT impairs epithelial barrier integrity and promotes the release of enzymes such as MMPs, thereby contributing to airway wall damage and remodelling (Khalili-Tanha et al., 2025). This process is finely regulated by multiple cytokines and signalling pathways, including TGF-β, platelet-derived growth factor (PDGF), fibroblast growth factor, epidermal growth factor, VEGF, CXCL2, CXCL3, IL-8/CXCL, Wnt signalling, and Notch pathways, which collectively drive the initiation and progression of EMT (Halwani et al., 2011; Kardas et al., 2020).

#### 4.1.3 Basement membrane thickening

Basement membrane thickening is another major feature of airway remodelling. In asthma, myofibroblasts accumulate in the subepithelial region through several mechanisms, including fibroblast activation and other stromal cell transitions (van Rijt et al., 2011). Under the influence of mediators such as TGF-β, these cells produce extracellular matrix components including collagen and fibronectin, leading to subepithelial fibrosis, airway narrowing, and reduced airway compliance (Berair and Brightling, 2014; Johnson et al., 2011).

#### 4.1.4 Increased vascularisation and remodelling of the airway wall

Vascular remodelling is an important component of airway remodelling in asthma. It includes neovascularisation, dilation of existing vessels, and increased vascular permeability, all of which facilitate inflammatory cell infiltration and mediator release (Gomulka et al., 2019b). Vascular endothelial growth factor (VEGF) acts as a key regulator of angiogenesis by promoting endothelial cell proliferation, neovascularization, and increased vascular permeability (Gomulka et al., 2019a; Hoshino et al., 2001). It interacts with inflammatory mediators such as MMP9, TGF-β, and IL-9 to accelerate inflammatory spread (Broide, 2008; Lee et al., 2006). Increased vascular leakage further contributes to airway wall oedema, narrowing, and impaired ventilation (Corrigan et al., 2011).

#### 4.1.5 Increased mucus secretion

Increased mucus secretion is a characteristic feature of airway remodelling. Under chronic inflammatory stimulation, especially in goblet cells, airway epithelial cells produce excess mucus that compromises airway patency (Pangeni et al., 2023; Siddiqui et al., 2021). Furthermore, alterations in the composition and structure of the extracellular matrix (ECM), particularly adjustments in components such as hyaluronic acid and proteoglycans, indirectly contribute to increased mucus viscosity and retention. This further impedes normal airway function (Descalzi et al., 2007).

#### 4.1.6 Chronic airway inflammation

Airway inflammation is closely linked to airway remodelling in asthma (Hough et al., 2020). Persistent inflammation promotes the release of Th2 cytokines such as IL-4, IL-5, and IL-13, contributing to subepithelial fibrosis, smooth muscle cell proliferation, and eosinophil infiltration (Conde et al., 2021). Chemokines such as CCL11 and CCL2 further recruit inflammatory cells into the airway, where they release mediators that aggravate oedema, mucus secretion, and structural remodelling (Ferreira et al., 2022; Ji and Li, 2023).

#### 4.1.7 Imbalance in immune response

Under normal physiological conditions, airway immune cells such as innate lymphoid cells (ILCs), T cells (CD4^+^ and CD8^+^), and myeloid cells collaborate to maintain airway homeostasis (Porsbjerg et al., 2020). In asthma, this equilibrium is disrupted, with abnormal Th2-type immune activation contributing not only to airway inflammation but also, in some contexts, to remodelling-related structural changes. Cytokines released by Th2 cells, including IL-4, IL-5, and IL-13, promote the activation of eosinophils, mast cells, and goblet cells within the airways (Frey et al., 2020). Inflammatory cell infiltration into the airway wall further increases the release of mediators such as histamine, prostaglandins, and TGF-β, which may promote airway remodelling by acting on structural cells including fibroblasts and smooth muscle cells (Xu et al., 2019). In addition, Th17-associated cytokines such as IL-17 and IL-22 may aggravate inflammatory and remodelling-related processes in the airway microenvironment (Loubaki et al., 2013).

### 4.2 Molecular mechanisms of airway remodelling

#### 4.2.1 TGF-β1

TGF-β1 is a multifunctional cytokine exhibiting both anti-inflammatory and pro-inflammatory effects, participating in asthma airway inflammation, immune responses, and airway remodelling processes (Shen et al., 2019). Previous studies suggest that TGF-β1 released by activated airway epithelial cells can activate Smad2/3 signalling in airway smooth muscle cells through paracrine effects, thereby promoting proliferation and collagen deposition and contributing to airway remodelling (Liu et al., 2019). Recent studies further reveal that the TGF-β1-activated Smad2/3 pathway specifically upregulates the expression of the transcription coactivator Yes-associated protein (YAP). As a key downstream factor driving the proliferation and migration of airway smooth muscle cells, YAP deepens our understanding of the TGF-β1-driven mechanism of airway remodeling (Zhao et al., 2023).

#### 4.2.2 SIRT1

Silent Information Regulator 2-associated Enzyme 1 (SIRT1) is widely expressed in various human tissue cells. It regulates gene transcription and target protein activity through acetylation, participating in processes such as ageing and metabolism (Brooks and Gu, 2009). SIRT1 deficiency exacerbates airway inflammation and asthma-related remodelling, whereas SIRT1 activation suppresses inflammatory cell infiltration, cytokine production, airway mucus secretion, and pathological collagen deposition in asthmatic mice (Huang et al., 2022). In an ovalbumin (OVA)-induced mouse asthma model, downregulation of SIRT1 expression reduces the content of the homologous loss-of-function phosphatase tensin-related protein gene (PTEN) on chromosome 10, whereas SIRT1 activation promotes PTEN upregulation and inhibits airway remodelling (Chen et al., 2015). Furthermore, the copper-peptide glycine-histidine-lysine tripeptide complex with copper can upregulate SIRT1 expression and activity, inhibit the TGF-β1/Smad pathway, and improve airway remodelling in asthmatic mice (Zhang Q. et al., 2023).

#### 4.2.3 YKL-40

Chitinase-like protein 3-like 1 (YKL-40) is an inflammatory protein closely associated with airway remodeling in asthma, primarily participating in this process by regulating the chemotaxis, adhesion, and migration of inflammatory cells (Konradsen et al., 2013). Elevated YKL-40 levels are observed in asthma patients and correlate with disease severity, subepithelial basement membrane thickening, and impaired lung function (Kim et al., 2024). YKL-40 enhances IL-8 production in bronchial epithelial cells by activating the C-Jun N-terminal kinase (JNK), extracellular signal-regulated kinase (ERK), and NF-κB pathways, thereby inducing proliferation and migration of airway smooth muscle cells (ASMCs). Furthermore, YKL-40 promotes ASMC proliferation and migration by activating localised focal adhesion kinase/scattered-expression classifier/Akt and MAPK pathways, thereby facilitating the accumulation of airway smooth muscle mass and exacerbating airway remodelling in asthma (Sun et al., 2020).

#### 4.2.4 ADAM33

Adhesin-dissociating metalloproteinase 33 (ADAM33), a member of the ADAM family, is primarily expressed in airway smooth muscle cells (ASMCs). Its activity influences ASMC function and is associated with cell proliferation and injury, having been identified as an asthma susceptibility gene (Sleziak et al., 2024). Available evidence suggests that ADAM33 participates in airway remodelling through multiple mechanisms, including effects on airway epithelial plasticity, fibroblast differentiation and proliferation, airway smooth muscle behaviour, and airway neovascularisation (Tripathi et al., 2014; Zhou et al., 2019).

#### 4.2.5 MIF

Macrophage Inhibitory Factor (MIF) is an immunomodulatory cytokine exhibiting chemotactic-like activity. It promotes inflammatory responses, directs cell migration, antagonises glucocorticoids, inhibits apoptosis, and stimulates the release of pro-inflammatory cytokines (Luo et al., 2021). MIF activates T cells and macrophages, increasing the production of pro-inflammatory mediators, disrupting ECM homeostasis, upregulating MMP-9 and MMP-12, whilst simultaneously reducing the activity of matrix metalloproteinase inhibitors. This may lead to excessive degradation of collagen and elastin, thereby promoting ASMC proliferation, migration, and airway structural injury (Calandra and Roger, 2003; Qu et al., 2009; Churg et al., 2003). MIF promotes autophagy in airway smooth muscle cells via its receptor CD74, thereby driving their proliferation, migration, and collagen deposition, ultimately leading to airway remodeling and structural damage in asthma (Li et al., 2020).

#### 4.2.6 IL-22

IL-22 is significantly expressed in children with asthma. Upon binding to its receptor, it activates the Janus kinase (JAK)/signal transducer and activator of transcription (STAT) pathway, stimulating fibroblast proliferation and collagen secretion. Concurrently, it activates neutrophils, enhances TGF-β-induced epithelial-mesenchymal transition (EMT) in bronchial epithelial cells, exacerbates airway inflammation, and promotes airway remodelling (He et al., 2022).

#### 4.2.7 Chemokines

**FIGURE 2:**
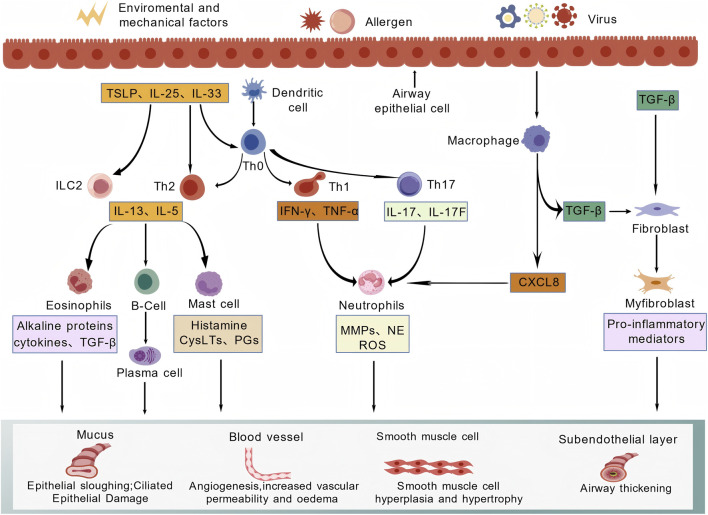
Simplified overview of inflammatory and structural mechanisms contributing to airway remodelling in paediatric asthma.

Eosinophil activation factor (Eotaxin) and CXCL2 belong to the chemokine family. Elevated levels recruit inflammatory cells such as eosinophils into the airways, exacerbating the inflammatory response. Prolonged inflammatory stimulation leads to airway epithelial damage and structural alterations including airway smooth muscle hyperplasia, resulting in airway remodelling (Bignold et al., 2022). Lin et al. (2022) proposed that mepolizumab can specifically antagonise IL-5, thereby inhibiting eosinophil activation in asthma patients, reducing inflammatory cell counts, and lowering complication risks. This consequently alleviates airway inflammation and suppresses airway remodelling. Furthermore, key targets including insulin-like growth factor-like peptide receptor 1, IL-17, TNF-α, Sema3E, and follistatin-like protein 1 all play significant roles in the process of airway remodelling in asthma (Lee et al., 2014; Liu et al., 2017; Margelidon-Cozzolino et al., 2022). The molecular mechanisms of airway remodelling in childhood asthma are illustrated in Figure 2.

Environmental, mechanical, allergen-related, and viral stimuli acting on the airway epithelium may trigger the release of epithelial alarmins and other mediators, which in turn influence innate and adaptive immune responses. The figure summarises interactions among epithelial cells, dendritic cells, T-helper cell subsets, macrophages, eosinophils, neutrophils, mast cells, fibroblasts, and myofibroblasts, together with representative cytokines and mediators implicated in remodelling-related processes. Downstream consequences include epithelial injury, mucus hypersecretion, angiogenesis and vascular leakage, airway smooth muscle hyperplasia/hypertrophy, and subepithelial thickening. This figure is intended as a simplified conceptual overview of upstream inflammatory and profibrotic inputs and their downstream remodelling-related effects, rather than a detailed receptor-level signalling map. Created with BioGDP.com.

### 4.3 Regulatory signalling pathways in airway remodelling

#### 4.3.1 PI3K/Akt signalling pathway

Activation of the PI3K/Akt signalling pathway constitutes a pivotal event in asthma-related airway remodelling. Akt inhibits glycogen synthase kinase phosphorylation, increases cyclin D1 expression, and promotes DNA replication, thereby inducing airway smooth muscle cell (ASMC) proliferation and phenotypic transformation. Akt activation promotes the expression of multiple genes associated with cell proliferation, migration, and differentiation, such as growth factor receptors and MMPs, which play pivotal roles in airway remodelling (Lim et al., 2009). Concurrently, the PI3K/Akt pathway enhances fibroblast activation and the synthesis and deposition of the extracellular matrix (ECM), thereby exacerbating airway fibrosis (Xie et al., 2019). Moreover, this signalling pathway participates in regulating key processes in vascular endothelial cells, including proliferation, migration, and lumen formation, thereby promoting the formation of new blood vessels in the airways and providing essential nutritional support for airway remodelling (Zhao et al., 2018).

#### 4.3.2 NF-κB signalling pathway

NF-κB is a crucial transcription factor playing a central role in airway remodelling during asthma, influencing the phenotypic transformation of smooth muscle cells and regulating their proliferation, differentiation, and other function (Sun et al., 2020; Song et al., 2020). Furthermore, NF-κB extensively participates in asthma pathogenesis through diverse molecules including growth factors, cytokines, inflammatory mediators, eosinophil chemotactic factors, adhesion molecules, and MMPs, forming a complex regulatory network within asthma and airway remodelling (Barbosa et al., 2023).

#### 4.3.3 TGF-β1/Smads signalling pathway

The TGF-β1/Smads signalling pathway plays a pivotal role in airway remodelling, particularly concerning the epithelial-mesenchymal transition (EMT) process (Yao and Fu, 2021). As a core regulatory factor, TGF-β1 induces epithelial-to-mesenchymal cell conversion, activates myofibroblasts, and accelerates extracellular matrix accumulation, thereby driving airway remodelling in the early stages of asthma pathogenesis. Moreover, recurrent episodes of airway inflammation constitute another pivotal factor promoting airway remodelling. Increased eosinophil counts elevate TGF-β1 expression levels, thereby further accelerating the EMT process and exacerbating airway remodelling phenomena (Quan et al., 2024).

#### 4.3.4 MAPK signalling pathway

The MAPK pathway constitutes a crucial intracellular signalling cascade involved in a range of cellular physiological activities including growth, development, differentiation, and apoptosis (Liang and Yang, 2019). This pathway primarily comprises three classical branches: ERK, JNK, and p38 MAPK. Research indicates that the ERK and JNK pathways positively regulate TGF-β1-induced growth in airway smooth muscle cells (Xie et al., 2007). p38 MAPK also regulates airway inflammation and remodelling induced by ASMCs. The MAPK pathway and its subfamilies participate in modulating TGF-β1 expression in asthma airway epithelium. TGF-β1’s pro-fibrotic effects influence airway remodelling by promoting extracellular matrix deposition, a process requiring the coordinated action of the MAPK pathway (Pelaia et al., 2003).

#### 4.3.5 JAK/STAT signalling pathway

The JAK/STAT pathway plays a crucial role in the expression of multiple cytokines and adhesion molecules in asthma (Lyu et al., 2024). In asthma, the JAK/STAT signalling pathway is primarily activated by cytokines such as IL-13 and IL-4. These cytokines induce a Th2-dominant response by activating the JAK/STAT pathway, thereby promoting characteristic features of asthma including immunoglobulin E production, bronchial hyperresponsiveness, airway remodelling, and mucus metaplasia (Junttila, 2018). Elevated inflammatory cytokine expression in asthma patients activates the JAK/STAT pathway, leading to excessive airway nitric oxide synthase production and increased nitric oxide levels. This exacerbates airway hyperresponsiveness and inflammatory responses, promotes tissue damage, and ultimately drives airway remodelling (Escamilla-Gil et al., 2022).

#### 4.3.6 Notch signalling pathway

The Notch signalling pathway is a highly conserved transduction pathway that plays a crucial regulatory role in cellular inflammation, immune imbalance, and subepithelial fibrosis. Notch1 deficiency is associated with the pathogenesis of pulmonary diseases such as chronic obstructive pulmonary disease (COPD), asthma, and pulmonary fibrosis (Antar et al., 2024). Research has revealed markedly elevated expression of Notch1 and Split hairy enhancer-like 1 (Hesl) proteins in the lung tissue of asthmatic mice. Treatment with the Notch signalling pathway inhibitor KyoT2 effectively mitigated airway epithelial fibrosis in these mice via a Hesl-dependent mechanism (Hu et al., 2015). Blocking the Notch signaling pathway (e.g., using anti-Notch4 antibodies) effectively reduces airway inflammation, hypermucus secretion, and airway hyperresponsiveness in asthma (Xia et al., 2018).

#### 4.3.7 Wnt/β-catenin signalling pathway

The Wnt/β-catenin signalling pathway plays a pivotal role in numerous biological developmental processes and disease states, exerting significant influence on the proliferation and differentiation functions of airway smooth muscle cells (ASMCs). During airway remodelling, this pathway participates in disease onset and progression by regulating cellular proliferation, differentiation, and the synthesis and degradation of the extracellular matrix (ECM) (Jia et al., 2021). Studies indicate that long-term asthma mouse models exhibit upregulation of Wnt protein and β-catenin expression in lung tissue, consistent with airway remodelling characteristics. Intervention using β-catenin-specific siRNA effectively mitigates airway inflammation and remodelling in asthmatic mice (Aros et al., 2021). Furthermore, in chronic asthma models, inhibiting β-catenin expression suppresses smooth muscle hyperplasia by blocking the tenascin C/platelet-derived growth factor receptor pathway (Hussain et al., 2017).

#### 4.3.8 Rho/ROCK signalling pathway

**FIGURE 3:**
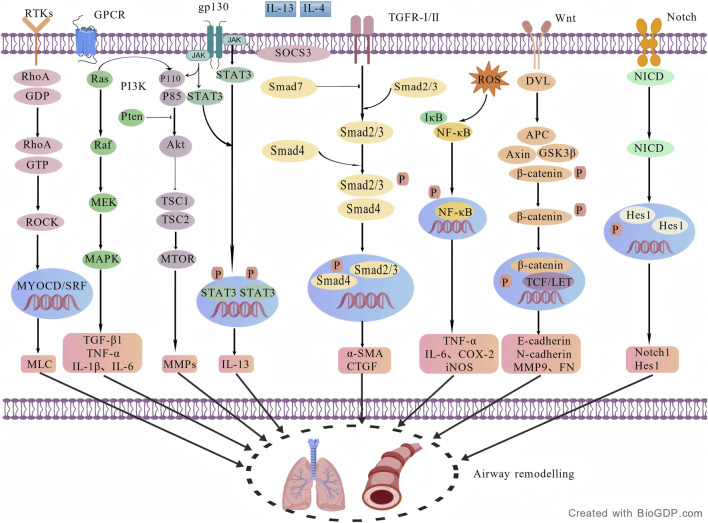
Major signalling pathways implicated in airway remodelling in paediatric asthma.

The Ras homologue (Rho)/Rho-associated coiled-coil protein kinase (ROCK) signalling pathway constitutes a crucial intracellular signal transduction pathway involved in regulating cytoskeletal remodelling and the generation of intracellular forces (Yang and Shi, 2021). Research indicates that activation of the Rho/ROCK pathway not only promotes the proliferation and hypertrophy of airway smooth muscle cells (ASMCs), but also influences processes such as migration, proliferation, and differentiation within the airway epithelium. Furthermore, by regulating the migration, adhesion, and activation of inflammatory cells, it exacerbates their infiltration and activation within the airways, thereby further intensifying the degree of airway remodelling (Zhang et al., 2020). The molecular mechanisms of signalling pathways involved in airway remodelling in childhood asthma are illustrated in Figure 3.

The diagram summarises representative pathways reported to participate in airway remodelling, including PI3K/Akt, NF-κB, TGF-β/Smads, MAPK, JAK/STAT, Wnt/β-catenin, Notch, and Rho/ROCK signalling. These pathways are shown as a simplified overview of remodelling-related intracellular signalling rather than a complete receptor-level map, and are intended to illustrate how upstream cytokine or growth factor inputs may converge on processes such as epithelial–mesenchymal transition, extracellular matrix deposition, airway smooth muscle proliferation and migration, mucus hypersecretion, and inflammatory amplification (Created with BioGDP.com).

## 5 EV-associated non-coding RNAs in paediatric asthma airway remodelling

EV-associated non-coding RNAs may regulate airway remodelling through a multi-level network. After transfer to target cells, including airway epithelial cells, smooth muscle cells, and immune cells, they may influence pathways such as TGF-β/Smad, Wnt/β-catenin, JAK/STAT, PI3K/Akt, ErbB, and FOXO1, thereby affecting EMT, ASMC proliferation and migration, ECM deposition, mucus hypersecretion, and chronic inflammation. However, the currently available evidence is heterogeneous. Stronger mechanistic studies generally include one or more of the following: direct isolation and characterization of EV cargo, EV uptake or tracing experiments in recipient cells, gain- and loss-of-function manipulation of candidate ncRNAs, and demonstration of downstream signalling or phenotypic effects after EV transfer. By contrast, many published studies describe remodelling-related functions of ncRNAs without directly demonstrating EV-mediated intercellular delivery. In addition, the mechanistic literature is currently dominated by miRNA-related studies, while direct evidence involving EV-associated lncRNAs and circRNAs in paediatric asthma airway remodelling remains limited. The following sections therefore distinguish, where possible, between direct EV-associated evidence and broader mechanistic ncRNA context. Accordingly, the discussion below focuses primarily on remodelling-relevant ncRNA findings that are most informative for interpreting EV-associated mechanisms, rather than providing an exhaustive overview of general airway remodelling biology. Given the limited number of paediatric studies directly demonstrating EV uptake, ncRNA transfer, and recipient-cell functional consequences, some evidence discussed below should be interpreted as mechanistic support rather than definitive proof of EV-dependent causality.

### 5.1 Extracellular vesicle non-coding RNAs and airway epithelial remodelling

EV-associated non-coding RNAs may regulate inflammation and fibrosis in airway epithelial cells and thereby influence epithelial-mesenchymal transition (EMT), although the strength of evidence for direct EV-mediated transfer differs across studies. In a cell-based EV-related epithelial model, miR-26a-5p was reported to target PTGS2 within the PGE2 signalling pathway, thereby reducing ferroptosis and inflammation in airway epithelial cells and suppressing EMT (Liu et al., 2023). This provides a novel therapeutic strategy for airway remodelling. Guo et al. (2022) reported that miR-23b-3p overexpression promoted EMT-related remodelling changes in an asthma model by suppressing epithelial markers such as epithelial cadherin and tight junction protein 1, whilst upregulating mesenchymal markers such as N-cadherin. Although these findings support a remodelling-related role of miR-23b-3p in airway epithelial cells, EV-mediated transfer was not directly demonstrated in that study. Tang et al. (2022) demonstrated in an experimental model that M2 macrophage-derived EVs carrying miR-30b-5p inhibited IRF7-related pyroptosis in airway epithelial cells and alleviated airway inflammation and fibrosis, thereby mitigating airway remodelling. Representative EV-associated and remodelling-related ncRNA candidates implicated in paediatric asthma airway remodelling are summarised in Table 1.

**TABLE 1:** Representative EV-associated and remodelling-related ncRNA candidates in paediatric asthma airway remodelling.

EV-associated or remodelling-related ncRNA	Target/Signal pathway	Primary molecular mechanisms	Effects on airway remodelling	Evidence level	Clinical relevance/Limitation
miR-26a-5p	PTGS2/PGE2 signalling pathway	Inhibits PTGS2/PGE2, alleviates ferroptosis and inflammation, and suppresses EMT	Inhibit	EV-related epithelial model	Candidate therapeutic target; direct EV-mediated transfer not fully validated
miR-23b-3p	Epithelial cadherin, desmocollin 1, N-cadherin	Downregulate epithelial markers, upregulate mesenchymal markers, and promote EMT	Promote	Mechanistic context only	Remodelling-related candidate; EV-mediated transfer not directly shown
miR-30b-5p	IRF7 signalling pathway	Inhibit pyroptosis, downregulate IRF7, and alleviate inflammation and fibrosis	Inhibit	Direct EV-associated evidence	Candidate therapeutic target; further paediatric and *in vivo* validation needed
miR-188	JARID2/Wnt/β-catenin pathway	Targeting JARID2 inhibits the Wnt/β-catenin pathway, thereby suppressing the proliferation of ASMCs	Inhibit	Mainly animal-model/mechanistic evidence	Potential therapeutic target; EV-mediated delivery not directly demonstrated
miR-133a	AKT phosphorylation	Inhibits AKT phosphorylation, reduces ASMC proliferation, and promotes apoptosis	Inhibit	Mechanistic context only	Potential therapeutic target; non-EV evidence and limited translational validation
miR-21-5p	PTEN/PI3K/AKT pathway	Inhibition of PTEN activates the PI3K/AKT pathway, promoting the proliferation and migration of ASMCs	Promote	Mechanistic context only	Candidate biomarker or target; EV-mediated transfer not specifically examined
miR-26a-5p	MMP, phosphodiesterase 7A	Promotes the expression of collagen I and other factors, thereby exacerbating fibrosis	Promote	Mainly model-based evidence	Context-dependent remodelling relevance; direct EV evidence remains limited
miR-326	Smad3/TGF-β pathway, MMP, cyclin	Inhibition of Smad3 attenuates TGF-β signalling, thereby reducing extracellular matrix synthesis and deposition	Inhibit	Mechanistic context only	Potential anti-remodelling target; direct EV-mediated delivery not demonstrated
miR-19a	PRMT1	Inhibition of PRMT1 promotes protein methylation and ECM synthesis when its expression is low	Promote	Adult/mechanistic evidence	Mechanistically relevant candidate; paediatric-specific and direct EV evidence limited
miR-19	MIF/JAK/STAT pathway	Activate MIF to promote Th2 differentiation and inflammatory responses	Promote	Mechanistic context only	Inflammation-related candidate; evidence mainly pathway-based rather than direct EV transfer
miR-378a-3p	ErbB signalling pathway	Promote the proliferation and resistance to apoptosis of ASMCs, and increase ECM proteins	Promote	Paediatric mechanistic context	Candidate biomarker; direct EV-mediated delivery not demonstrated
miR-130b-5p	FOXO1	Modulates FOXO1, reduces Th17 cells, alleviates inflammation	Inhibit	Mechanistic context only	Immune-regulatory candidate; EV-mediated transfer not specifically examined
miR-15/16	IL-7 receptor α-chain/IL-7 signalling pathway	Maintains Treg function; when deficient, disrupts immune tolerance and exacerbates inflammation	Promote	Animal-model mechanistic evidence	Candidate marker of immune dysregulation; deficiency model does not establish EV-mediated transfer

The studies summarised in this table differ in the level of evidence supporting direct EV-mediated transfer. Some entries are based on direct EV-associated findings, whereas others are included as remodelling-related ncRNA, mechanisms relevant to EV, biology.

### 5.2 Remodelling of airway smooth muscle by EV-associated non-coding RNAs

EV-associated ncRNAs and remodelling-related ncRNA mechanisms may influence the proliferation and apoptosis of airway smooth muscle cells through regulation of cell-cycle and growth-factor signalling pathways. Shan et al. (2022) utilised TGF-β1-treated cells and an OVA-induced mouse asthma model to identify miR-188 as a remodelling-related regulator targeting JARID2 and the Wnt/β-catenin pathway. Although EV-mediated delivery was not directly demonstrated, these findings provide mechanistic context linking miR-188 to ASMC proliferation, mucus secretion, and collagen deposition. miR-133a has also been reported to regulate the proliferation and apoptosis of smooth muscle cells under TGF-β1 stimulation by inhibiting AKT phosphorylation, thereby suppressing ASMC proliferation and promoting apoptosis (Shao et al., 2019). However, this study did not directly demonstrate EV-mediated transfer, and is therefore discussed here as remodelling-related mechanistic context. Similarly, miR-21-5p has been reported to stimulate ASMC proliferation and migration by suppressing PTEN and enhancing PI3K/AKT-related signalling (Xiao et al., 2023), although direct EV-mediated delivery was not specifically examined.

### 5.3 EV-associated non-coding RNAs and ECM remodelling

EV-associated non-coding RNAs influence ECM protein synthesis and degradation by regulating gene expression of MMPs and other factors, thereby affecting matrix remodelling and degradation. Zhong et al. (2022) demonstrated that miR-26a-5p targets and binds to non-coding regions of genes including MMP and phosphodiesterase 7A, promoting collagen I and alpha-smooth muscle actin expression. Inhibiting miR-26a-5p expression, conversely, increased apoptosis rates in asthma model cells while downregulating inflammatory cytokines and fibrosis markers, thereby suppressing airway remodelling processes. These findings suggest that miR-26a-5p may be involved in airway remodelling in asthma. In non-EV mechanistic studies, miR-326 has been reported to target key molecules in the TGF-β signalling pathway, such as Smad3, which plays a central role in TGF-β-induced ECM accumulation and cell proliferation. By suppressing Smad3 expression, miR-326 may reduce TGF-β-induced synthesis of type I collagen and fibronectin (Kang, 2017). Additionally, miR-326 has been reported to inhibit ASMC proliferation and ECM deposition by targeting genes associated with ECM synthesis, such as MMPs and cyclin genes, thereby exerting a protective role in airway remodelling (Usman et al., 2021). However, direct EV-mediated delivery was not demonstrated in these studies, and they are therefore discussed here as mechanistic support for a remodelling-related role of miR-326. Sun et al. reported that miR-19a is downregulated in airway smooth muscle cells from adult asthma patients (Sun et al., 2017), and that miR-19a suppresses PRMT1 expression by directly targeting its mRNA untranslated region. When miR-19a expression decreases, PRMT1 expression increases, promoting protein methylation and enhancing the synthesis and deposition of type I collagen and fibronectin. These changes may contribute to ASMC proliferation and migration, airway wall thickening, and airway remodelling. Similarly, the observed effects of miR-19a on PRMT1 and ECM synthesis support a remodelling-related mechanism, but not specific proof of EV-mediated transfer.

Notably, miR-26a-5p appears to show context-dependent effects across different experimental settings. In an epithelial injury-related EV model, it was associated with reduced ferroptosis, inflammation, and EMT, whereas in other remodelling-related models it has been linked to fibrosis-associated pathways and increased collagen-related responses. These apparently divergent findings may reflect differences in cell type, disease model, and downstream signalling context. At present, miR-26a-5p should therefore not be considered uniformly protective or pathogenic in airway remodelling.

### 5.4 EV-associated non-coding RNAs in airway inflammation and immune regulation

EV-associated non-coding RNAs may influence the balance of inflammatory cells and lymphocyte subsets in the airway, in part through the regulation of mediators such as MIF and eotaxin. Thymic interstitial lymphocyte-derived factor, as a lymphocyte growth factor, drives Th2 differentiation by activating the JAK/STAT signaling pathway, thereby triggering inflammatory responses (Soumelis et al., 2002; Rochman et al., 2010). In Th2-mediated inflammation, miR-19 synergistically interacts with MIF to amplify the JAK/STAT signaling pathway, thereby exacerbating the immune response (Yang et al., 2022; Simpson et al., 2014). Meanwhile, Th2 expression activates immune cells to produce excessive reactive oxygen species, reduces antioxidant enzyme expression, and subsequently suppresses the antioxidant defense system. This exacerbates airway inflammation and oxidative stress, thereby intensifying airway remodeling (Rangasamy et al., 2005; Zhu et al., 1999). Wong et al. identified miR-378a-3p in a paediatric asthma-related context and linked its expression to remodelling-associated features and ErbB-related signalling (Wong et al., 2024). However, this study did not directly demonstrate EV-mediated delivery, and is therefore interpreted as paediatric mechanistic context rather than direct evidence of EV-dependent transfer.

**FIGURE 4:**
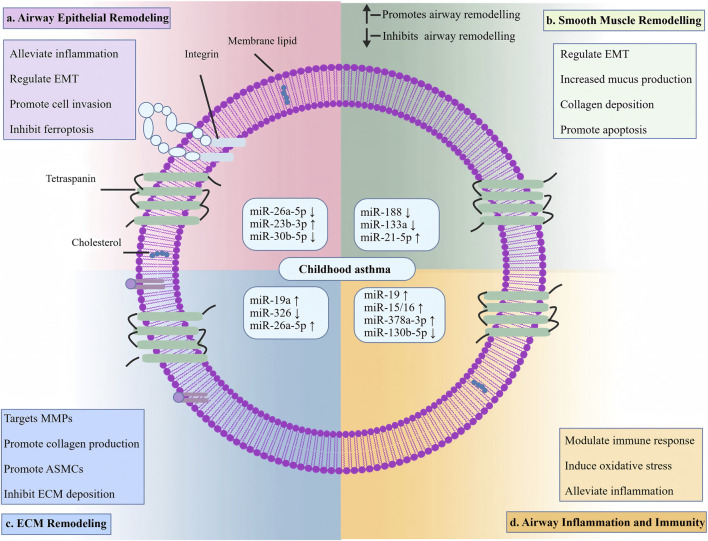
Multi-level regulatory network of EV-associated non-coding RNAs in paediatric asthma airway remodelling.

Research suggests that miR-130b-5p may bind to the 3′untranslated region of forkhead box transcription factor O1 (FOXO1), thereby affecting FOXO1 stability and translation (Zhu et al., 2020). This mechanism reduces the proportion of helper T (Th) 17 cells, thereby alleviating airway inflammation and regulating immune balance, although EV-mediated transfer was not specifically examined (Malik et al., 2017). Additionally, certain EV-associated non-coding RNAs exhibit anti-inflammatory and pro-inflammatory effects (Li et al., 2019). Johansson et al. (2023) employed OVA as an allergen to establish a mouse model simulating childhood asthma. Mice deficient in miR-15/16 within regulatory T (Treg) cells exhibited greater airway eosinophil infiltration compared to controls. Subsequent investigations revealed that miR-15/16 deficiency increased IL-7 receptor alpha chain expression, enhancing IL-7 signalling pathways and downstream gene expression, thereby impairing Treg function. These findings suggest that loss of miR-15/16 may disrupt immune tolerance and amplify inflammatory responses, thereby contributing to airway remodelling; however, they do not by themselves establish EV-mediated intercellular delivery. The mechanism by which extracellular vesicles regulate non-coding RNA in airway remodelling during childhood asthma is illustrated in Figure 4.

The central EV schematic represents intercellular transfer of EV-associated ncRNA cargo. The four surrounding panels summarise reported associations of EV-associated ncRNAs with airway epithelial remodelling, airway smooth muscle remodelling, extracellular matrix remodelling, and airway inflammation/immunity. The listed ncRNAs and arrows indicate the general direction of reported regulatory effects in the cited literature, with upward arrows indicating promotion and downward arrows indicating inhibition of remodelling-related processes. This schematic is intended as an integrative summary linking EV-associated ncRNAs, target cell types, representative biological effects, and airway remodelling outcomes, rather than a direct mechanistic map for every individual ncRNA (Created with BioGDP.com).

## 6 Conclusion and outlook

As a prevalent chronic respiratory disease, asthma exhibits complex pathogenesis, with airway remodelling serving as a pivotal pathological process underpinning its persistent progression and exacerbation. This paper summarises the morphological mechanisms of airway remodelling in paediatric asthma, encompassing chronic airway inflammation, airway smooth muscle cell proliferation and migration, airway epithelial-to-mesenchymal transition, basement membrane thickening, increased and remodelled airway wall vascularisation, heightened mucus secretion, and dysregulated immune responses. Molecular biological mechanisms underpinning paediatric asthma airway remodelling include TGF-β1, SIRT1, YKL-40, ADAM33, MIF, IL-22, and chemokines. Furthermore, the regulatory signalling pathways governing airway remodelling in childhood asthma primarily involve PI3K/Akt, NF-κB, TGF-β1/Smads, MAPK, JAK/STAT, Notch, Wnt/β-catenin, and Rho/ROCK. In a subset of patients, particularly those with persistent or severe disease, these processes may contribute to relatively fixed structural airway changes. As an emerging therapeutic modality, EV-associated non-coding RNAs demonstrate significant potential in regulating cellular behaviour, material transport, and signal transduction. This paper summarises the regulatory mechanisms of EV-associated non-coding RNA in airway remodelling during childhood asthma, encompassing remodelling of the airway epithelium, airway smooth muscle, extracellular matrix (ECM), airway inflammation, and immune modulation.

Currently, although research into airway remodelling in childhood asthma has preliminarily revealed its complex pathological network and the potential regulatory role of EV-associated non-coding RNAs, certain limitations persist within this field. Most studies included in this review did not provide direct experimental evidence confirming the endosomal origin of vesicles, which limits their classification as exosomes. This aligns with MISEV recommendations to avoid biogenesis-specific terms unless substantiated. Furthermore, while many cited studies report associations between EV-associated ncRNAs and airway remodelling outcomes (such as EMT, ASMC proliferation, ECM deposition, or inflammation), they often rely on correlative data (e.g., expression changes or *in vitro* modulation) rather than direct demonstration of causality or functional EV-mediated delivery and uptake in recipient cells. Therefore, the mechanistic interpretations presented in this review should be considered suggestive rather than definitive causal proof. In addition, many available studies still focus on isolated pathological components and association-level observations, rather than integrated causal validation across EV transfer, ncRNA function, recipient-cell response, and remodelling outcome. Their precise causal role in driving airway remodelling and the intricate regulatory mechanisms involved still require further validation through rigorous functional studies. Furthermore, methods for isolating, purifying, and characterising extracellular vesicles remain unstandardised, with their inherent heterogeneity severely compromising data reproducibility and reliability. Concurrently, existing animal models possess inherent limitations in simulating the unique pathogenesis of childhood asthma—such as immune development and dynamic airway structural changes—thereby constraining the translation of basic research findings into clinical applications. The sensitivity and specificity of extracellular non-coding RNA as a non-invasive biomarker urgently require validation through large-scale, prospective clinical cohorts. From a clinical perspective, EV-associated ncRNAs are increasingly being discussed as potential biomarkers in asthma. Compared with established markers such as fractional exhaled nitric oxide (FeNO) and blood eosinophil counts, EV-associated ncRNAs may provide broader mechanistic information by reflecting intercellular communication and remodelling-related signalling. However, current evidence remains insufficient to define their diagnostic sensitivity, specificity, reproducibility, or incremental clinical value over existing biomarkers, particularly in paediatric populations. At present, they should therefore be regarded as promising candidate biomarkers rather than clinically validated diagnostic tools. Furthermore, RNA-based intervention strategies face numerous challenges, including *in vivo* delivery efficiency, stability, and potential off-target effects.

Another important challenge is the heterogeneity of EV-associated ncRNA profiles across sample sources. EVs isolated from serum, bronchoalveolar lavage fluid, sputum, or cell-culture supernatants may differ substantially in composition, cellular origin, and biological interpretation. In addition, age, treatment exposure, disease phenotype, disease severity, and methodological differences in EV isolation and characterisation may all affect the reported ncRNA signatures. These factors should be considered when comparing studies and evaluating translational potential.

Emerging evidence also suggests that EV-associated ncRNAs may play a role in regulating cellular metabolism, which is increasingly recognized as a critical driver of airway remodelling and immune dysregulation in asthma. For instance, EVs can transfer metabolites and ncRNAs that influence energetic metabolism in bronchial epithelial and smooth muscle cells, potentially linking metabolic reprogramming to enhanced ATP production, altered ciliary function, and pro-remodelling phenotypes. Further exploration of these metabolic-regulatory mechanisms could provide new insights into the complex interplay between EV-ncRNA signalling and airway pathology.

Future research may further investigate the specific mechanisms by which non-coding RNAs associated with extracellular vesicles influence airway remodelling in childhood asthma, particularly how microRNAs and long non-coding RNAs modulate the remodelling process through regulating relevant genes and signalling pathways. Additionally, investigations could explore utilising EV-associated non-coding RNAs as biomarkers to predict the progression and prognosis of childhood asthma, alongside developing novel therapeutic strategies based on these molecules to provide more effective treatments for paediatric asthma patients. Concurrently, in-depth research should focus on employing multi-omics technologies such as single-cell sequencing and spatial transcriptomics to systematically map the dynamic networks through which extracellular vesicle non-coding RNAs interact with key signalling pathways (e.g., TGF-β, Wnt) to co-regulate the behaviour of airway epithelial cells, smooth muscle cells, and immune cells. Functional validation of the causal roles of key molecules should be conducted using gene editing tools. The potential of engineered EV-based delivery systems also merits further investigation. By loading specific miRNA mimetics or inhibitors, these systems could enable precise intervention in airway remodelling processes. These studies hold promise for deepening our understanding of the pathogenesis of childhood asthma and providing novel approaches for clinical diagnosis and treatment.

Overall, current evidence suggests that EV-associated ncRNAs may participate in airway remodelling through effects on epithelial injury, smooth muscle behaviour, ECM turnover, and immune regulation. Interpretation of these findings should also take into account potential variability related to sample source, including serum, bronchoalveolar lavage fluid, and cell-culture systems, as well as confounding factors such as age, treatment status, and disease phenotype. However, the evidence base remains heterogeneous, and many cited studies describe remodelling-related ncRNA functions without directly demonstrating EV-mediated transfer. In addition, direct mechanistic evidence involving EV-associated lncRNAs and circRNAs in paediatric asthma airway remodelling remains sparse, with current literature being dominated by miRNA-related studies. Finally, much of the available evidence derives from adult samples, *in vitro* systems, or murine models, and further paediatric-specific validation will be essential before these molecules can be considered robust biomarkers or therapeutic targets.
